# A Review on Concrete Structural Properties and Damage Evolution Monitoring Techniques

**DOI:** 10.3390/s24020620

**Published:** 2024-01-18

**Authors:** Jinghua Zhang, Lisha Peng, Shuzhi Wen, Songling Huang

**Affiliations:** Department of Electrical Engineering, Tsinghua University, Beijing 100084, China; jinghua-20@mails.tsinghua.edu.cn (J.Z.); penglisha@mail.tsinghua.edu.cn (L.P.); wensz21@mails.tsinghua.edu.cn (S.W.)

**Keywords:** concrete, structural properties, damage evolution monitoring, structural health monitoring

## Abstract

Concrete structures have emerged as some of the most extensively utilized materials in the construction industry due to their inherent plasticity and high-strength characteristics. However, due to the temperature fluctuations, humidity, and damage caused by human activities, challenges such as crack propagation and structural failures pose threats to the safety of people’s lives and property. Meanwhile, conventional non-destructive testing methods are limited to defect detection and lack the capability to provide real-time monitoring and evaluating of concrete structural stability. Consequently, there is a growing emphasis on the development of effective techniques for monitoring the health of concrete structures, facilitating prompt repairs and mitigation of potential instabilities. This paper comprehensively presents traditional and novel methods for concrete structural properties and damage evolution monitoring, including emission techniques, electrical resistivity monitoring, electromagnetic radiation method, piezoelectric transducers, ultrasonic techniques, and the infrared thermography approach. Moreover, the fundamental principles, advantages, limitations, similarities and differences of each monitoring technique are extensively discussed, along with future research directions. Each method has its suitable monitoring scenarios, and in practical applications, several methods are often combined to achieve better monitoring results. The outcomes of this research provide valuable technical insights for future studies and advancements in the field of concrete structural health monitoring.

## 1. Introduction

Concrete, a widely employed construction material renowned for its excellent durability and impact resistance, has extensive applications in critical infrastructure such as buildings, bridges, tunnels, and dams, contributing to the establishment of secure and dependable living and working environments that hold paramount importance in our daily lives. However, due to its brittle nature, concrete is prone to structural cracking and failures caused by factors such as temperature variations, humidity, and changes in loading conditions, posing a threat to human safety and property [1]. Therefore, the implementation of regular inspections for concrete structures facilitate is necessary to extend their service life. Conventional inspection methods, such as visual or camera-based inspections [2,3], stress monitoring [4], and borehole inspection [5] are already extensively applied in engineering. Visual or camera-based inspections depend on human interpretation or image processing algorithms to analyze surface images of concrete structures, yet the efficacy is limited by the absence of real-time crack monitoring capabilities and the incapacity to detect internal cracks. Stress monitoring involves integrating sensors within concrete structures for the real-time assessment of stress levels. However, the intricate placement of sensors and restricted monitoring range make it challenging to effectively monitor large-scale concrete structures. Additionally, borehole inspection entails drilling holes on the surface of concrete structures to observe variations in internal cracks. While it is a high-precision testing method, it lacks real-time crack monitoring and involves a certain level of destructiveness [6], thus limiting the comprehensive assessment of concrete structure stability.

With a growing focus on ensuring the sustained safety of concrete structures throughout their lifespan, there has been an escalating demand for the implementation of structural health monitoring (SHM) techniques, replacing conventional non-destructive testing (NDT) methods [7]. The application of SHM techniques allows for the real-time acquisition of data related to the evolution of concrete cracks, enabling the monitoring of structural strains and deformations. This is crucial for understanding the behavior and changes occurring throughout the structure’s service life. The valuable information obtained plays a pivotal role in devising effective maintenance and preservation strategies, thereby preventing further deterioration and potential accidents.

As a brittle material similar to rock, concrete structural damage often comes with the release of various forms of energy such as sound, light, and electricity. Consequently, the novel approaches to concrete SHM predominantly revolve around the conversion of energy. These approaches employ diverse sensor types to convert energy variations during crack propagation into quantifiable signals, allowing for inferences about the health status of concrete structures. Reinforced concrete structures are one of the most common composite structural systems in buildings, primarily used in bridges and large residential constructions. However, the structural elasticity of reinforced concrete components tends to deteriorate over time, leading to issues such as aging, rebar corrosion, fatigue, and cumulative damage. The development of cracks in reinforced concrete is influenced by the material strength of concrete and rebar, the amount of rebar used, bonding characteristics, and component dimensions. Strength analysis and damage monitoring of such structures are critical research areas. Various methods, including acoustic emission monitoring [8,9], piezoelectric transducers monitoring [10,11,12,13,14], ultrasonic testing [15,16], and distributed optical fiber sensor monitoring [17,18], have been extensively studied. Traditional non-destructive testing methods like eddy current testing and magnetic field testing [19] are also applied for corrosion detection in rebar. Chen et al. [20,21] studied the application of NDT testing methods in reinforced concrete structure inspection. However, there is limited research on concrete structures with minimal reinforcement and plain concrete. Such structures have widespread applications in dams, tunnels, roads, and other areas, but comprehensive review studies on these structures are currently limited.

In recent years, numerous scholars have conducted some research in this area, with some offering concise summaries of previous works. For instance, Patryk et al. [7] provided an overview of the application of NDT methods in structural safety monitoring. Zheng et al. [22] investigated the application of NDT methods in concrete bridges. Vertrynge et al. [23] examined the application of acoustic emission (AE) technology in masonry structures. However, current research reviewing concrete SHM primarily focuses on NDT and AE techniques, lacking a systematic introduction of novel monitoring methods based on electromagnetic signals. The study of material properties contributes to the prevention of concrete cracking. Existing review articles lack a corresponding summary on the changes in concrete material properties and monitoring methods. Therefore, this paper focuses on concrete structural properties and damage evolution monitoring techniques, particularly emphasizing the electrical signal monitoring method. It systematically introduces various methodologies within the field of concrete SHM, covering AE, electrical resistivity (ER) monitoring, electromagnetic radiation (EMR) methods, ultrasonic testing (UT), piezoelectric transducers and infrared thermography (IRT) approach, elucidating their underlying mechanisms, applications, advantages, limitations, and future development directions.

## 2. Acoustic Emission Monitoring

### 2.1. Introduction and Principle of the AE Monitoring

**Figure 1:**
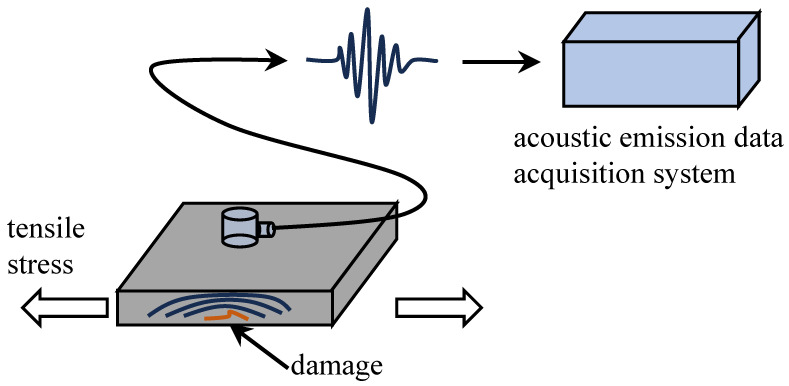
The principle of AE monitoring.

**Figure 2:**
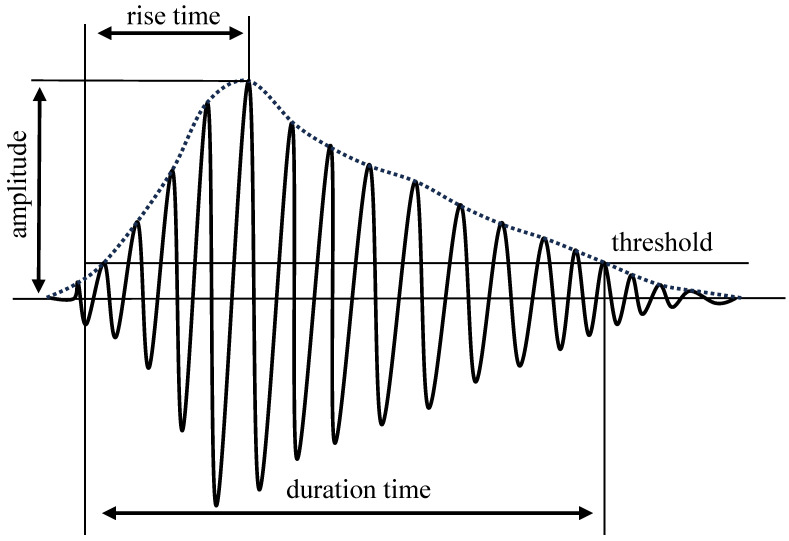
The AE signals.

Materials or structures undergo deformation or fracture when subjected to external or internal forces. The phenomenon in which the deformation energy is released in the form of elastic waves is known as AE. The basic principle of concrete crack AE monitoring is to capture the acoustic wave signals generated during concrete cracking using piezoelectric sensors and convert them into electrical signals for analysis and processing. The AE signals are closely associated with the vibration of the material. However, the material’s vibration is influenced by various factors. Researchers are placing more emphasis on studying the relationship between the signals and concrete properties rather than focusing on the explanation of the mechanisms. By analyzing the relationship between AE signals and applied stress during cracking, material dimensions, crack orientation, and other factors, relevant information regarding the safety of the concrete structure can be inferred. The principle of the AE monitoring method is illustrated in Figure 1. AE signals, as shown in Figure 2, exhibit an increase in oscillation amplitude during the rising time and decay in oscillation during the falling time. Among the signal characteristics of AE, energy and *b*-value are crucial parameters for assessing concrete damage. AE energy is defined as follows:

(1) $$\begin{array}{cc} E & =\int_{t_{1}}^{t_{2}}U^{2}dt, \end{array}$$

where *U* is the voltage of the AE signal, $t_{1}$ and $t_{2}$ are the start and end times of the AE signal. And the *b*-value is defined as

(2) $$\begin{array}{cc} \log_{10}N & =a-b\frac{A_{db}}{20} \end{array}$$

where $A_{db}$ is the peak amplitude of the AE signal in decibels, *N* is the number of AE hits of magnitude ≥ the sampling threshold.

In the earlier stages of AE development, substantial endeavors were focused on investigating the underlying principles of AE phenomena and examining the AE behavior in the deformation and fracture processes of diverse materials, such as wood and metal. Rusch et al. pioneered the use of AE to study the relationship between fracture process and volume change [24]. Due to the sensitivity of AE signals to crack propagation and structural instability, it has been currently a commonly used technique for estimating concrete structure properties and degree of damage. The key findings in the development of AE monitoring methods are shown in Table 1.

### 2.2. Structural Properties Estimation

**Figure 3:**
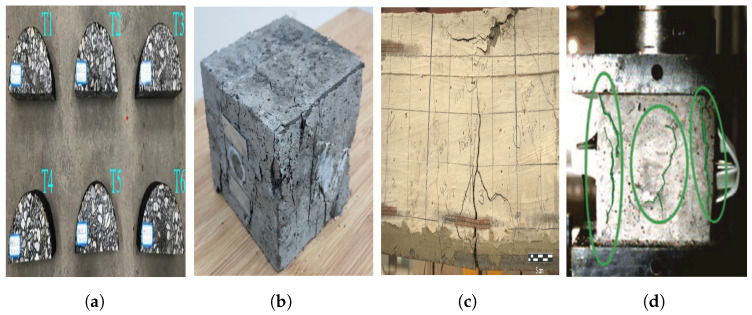
Applied to different types of concrete: (**a**) asphalt concrete [31], (**b**) rubber concrete [32] (Copyright 2021, Elsevier), (**c**) ultra-high-performance concrete [34] (Copyright 2021, John Wiley and Sons), and (**d**) slag concrete (the green circle represents the area of crack) [35] (Copyright 2020, Elsevier).

To enhance the performance and properties of concrete, admixtures are commonly incorporated into concrete mixes in varying proportions. These admixtures play a crucial role in altering the physical, chemical, and mechanical characteristics of concrete, thereby improving its strength, durability, crack resistance, and impermeability. With a close correlation between AE signals and concrete aggregates, noticeable variations in the structural behavior of concrete can be observed when different admixtures are utilized. Consequently, as shown in Figure 3, AE signals have been widely applied in estimating the structural strength of diverse concrete types, including asphalt concrete [31], rubber concrete [32], self-compacting concrete [33], ultra-high-performance concrete [34], and slag concrete [35].

The mechanical strength of admixed concrete displays inherent variability, and the characteristics of damage are influenced by the specific admixtures employed. AE signals could provide a valuable means to evaluate the extent of damage in admixed concrete, demonstrating remarkable sensitivity to forces acting on components such as fibers and reinforcing bars. This sensitivity enables the detection of damage in its early stages, even before visible signs become apparent [36]. The overall *b*-value, representing the ratio of small events to large events in AE signals, remains consistent across concrete specimens with varying fiber polymer densities. However, a rapid decrease in the *b*-value is observed during the yielding stage, which can serve as an indicator of concrete condition [37]. Furthermore, AE signals generated from the fracture of concrete with different levels of brittleness exhibit discernible variations, with the *b*-value in proximity to the stress drop gradually decreasing with an increasing brittleness degree [38].

Moreover, AE signals generated from concrete cracking are closely associated with factors such as concrete size, mode of force application, and tensile rate. These signals exhibit variations in concrete structures tested under different fracture conditions, including uniaxial compression [35], three-point bending tests [39], and the incorporation of silent cracking agents [40]. The relationship between AE signals and the stress level is nonlinear, with cumulative AE signals at peak stress showing nonlinear growth as concrete size increases [41]. Carpinteri et al. [42] demonstrated that the fracture energy dissipated per unit fracture area increases with an increase in sample size, while the energy detected by AE sensors shows an opposite trend. The direct correlation between the two forms of energy cannot be established. However, with the increase in loading rate, both concrete fracture energy and accumulated AE energy rise simultaneously [43]. The accumulation of AE energy serves as an indicator of fracture energy variations at different loading rates. The change in *b*-values allows for the analysis of different failure modes under various force applications. Yue et al. [44] investigated the AE signals of concrete under tensile failure and established an empirical relationship between strain and AE energy. Similarly, Prabhat et al. [45] studied AE characteristics of concrete under shear, shear flexure, and flexure failure and established the correlation between AE signals and loading methods in concrete beams through the average frequency and rise angle of AE signals.

### 2.3. Structural Damage Assessment

Building upon the foundation of AE signal analysis for estimation of structural properties, numerous scholars have examined the relationship between AE signals and concrete structural damage, establishing numerical models suitable for different scenarios.

Parameters such as energy, *b*-value, and T-value (product of *b*-value and AE signal density) play crucial roles in concrete damage research. The relationships between concrete surface damage variables, AE energy, and volume damage variables during fracture processes are established based on the correlation coefficient between applied work and AE energy [46]. Zhao et al. [47] investigated the evolution characteristics of AE signals in terms of amplitude, *b*-value, activity level, and frequency spectrum during concrete fracture, highlighting the continuous decline in *b*-values before failure. Ren et al. [39] found that the T-value is a more suitable criterion for evaluating the degree of damage in concrete beams compared to the *b*-value and constructed a fracture process zones using AE data.

Based on the correlation between AE signals and damage, some scholars have proposed AE models that provide interpretable insights into damage evolution. Nitin et al. [48] utilized wavelet entropy as a measure of spectral disorder to identify signals and estimate concrete damage. The statistical variance of wavelet entropy distributions increased with higher stress levels, indicating a fracture process involving multiple sources and mechanisms. Viet et al. [49] developed a classification model for damage stages using mean and standard deviation values of AE signal parameters, including counts, duration, amplitude, rise time, energy, rise angle, and average frequency. Vidya et al. [50] employed a probabilistic approach based on Gaussian mixture modeling to identify yield points using AE signals, comparing them with yield points obtained from plastic strain energy, thereby determining a damage index.

In recent years, there has also been a growing trend in combining image recognition methods with AE techniques to study the evolution of concrete cracking and damage. Giuseppe et al. [51] established a correspondence between concrete crack formation and propagation and AE signals using techniques such as AE, digital image correlation, and dynamic identification. Guo et al. [52] utilized AE and 3D digital image technology to measure internal AE signals and surface deformation features.

Fractal dimension and several AE analysis algorithms (denoising, time-frequency parameter analysis, start time detection, source localization, and characterization) have been applied to explain brittle failure mechanisms [53,54,55]. By analyzing the characteristics of AE signals, crack size and location in concrete structures can be determined. Charlotte et al. [54] proposed a hierarchical clustering algorithm based on cross-correlation, successfully distinguishing macro-cracking, corrosion-induced cracking, and micro-cracking during the corrosion process. The dissimilarity between the normal state, micro-cracks, and macro-cracks (fracture) in concrete beam specimens is distinguished using the k-nearest neighbor algorithm with an accuracy of up to 99.61% [56]. The reliability of two AE signal selection methods (Akaike criterion and fixed threshold) and four localization algorithms (adaptive meshing algorithm, genetic algorithm, globalized and bounded Nelder–Mead algorithms, and the simplex algorithm) in locating damage in concrete structures was also investigated [57].

AE, an advanced non-contact and real-time NDT technique, is commonly employed to identify and analyze structural instability and damage in critical civil engineering structures like power plants, bridges, and dams. It can be used to assess the properties of various concrete materials, aid in the analysis of stress conditions, and establish predictive models for the evolution of concrete damage. It also involves the analysis of time records, arrival time differences, energy levels, and amplitude variations of AE signals generated during concrete cracking, enabling the determination of crack initiation time, quantity, location, and activity. Nonetheless, certain challenges must be acknowledged and addressed to ensure the accuracy and reliability of AE monitoring. These challenges include environmental noise interference, optimization of sensor arrangement, and the complexity associated with signal analysis algorithms. Quantitative methods for complex concrete cracks are not yet mature, and separating the effects of multiple cracks is a direction for future development. Furthermore, the connection between AE signals and the progression of damage remains unclear, and there is currently limited research on the evolution and prediction of damage.

## 3. Electrical Resistivity Monitoring

### 3.1. Introduction and Principle of ER Monitoring

**Figure 4:**
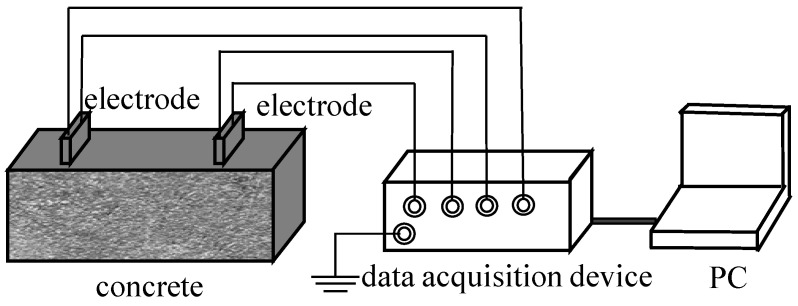
The basic schematic of ER method.

The ER is the ability of a material to impede the flow of electric current. The concrete ER can vary widely, ranging from 10 to $10^{5}$Ω·m, depending on factors such as moisture content and composite composition [58]. The ER method for assessing the safety of concrete structures is based on the movement of electrons caused by internal structural changes or applied loads, resulting in ER variations. The formation of double electric layers and ion flow within the concrete matrix serves as a significant source of electrical signals [59]. By studying the changes in ER, it becomes possible to infer the strength and extent of damage in concrete structures. Figure 4 is a basic schematic of the ERM method. By placing electrodes on the surface of concrete, electrical signals are collected and transmitted to a PC through a data acquisition device. Electrical conductivity is obtained according to Equation (3)

(3) $$\begin{array}{cc} \rho & =R_{c}\frac{A}{L}, \end{array}$$

where $R_{c}$ is the resistance data of concrete, *A* is the cross-sectional area, and *L* is the gauge length between the two electrodes.

The investigation of concrete ER could date back to the 1960s, and since the 1990s, there has been an increasing research focus on exploring the correlation between ER and the stability of concrete structures. Whittington et al. [60] delved into the relationship between concrete mix proportions, electrical properties of constituents, and concrete ER, confirming the variations in ER among different concrete materials. Building upon this, King and Luo et al. [61] further conducted laboratory uniaxial compression tests on concrete specimens and observed changes in resistance as the applied stress reached 20–90% of failure stress. The key findings in the development of ER monitoring methods are shown in Table 2. In recent research, the ER method has become a primary tool for analyzing the properties of concrete materials, and sensors are embedded within the concrete to monitor the evolution of damage.

### 3.2. Structural Properties Estimation

Concrete ER exhibits variations depending on the inclusion of different materials. Analysis of ER changes enables the determination of properties such as strength, durability, and impermeability [64]. Recent findings indicate that the integration of conducting components such as carbon fiber into cement produces a cement composite suitable for strain sensing. The preparation process of conductive concrete specimens and the methods of mounting electrodes were investigated [65]. Some researchers also identified the optimal carbon fiber doping level through ER variations and applied it to smart concrete [66]. Given the intricate composition of concrete material ratios and the influence of external environmental conditions, the evaluation of concrete structures heavily depends on practical on-site test results in engineering applications.

Consequently, models established for the assessment of concrete structures are often empirical rather than derived from strict theoretical principles. Le et al. [67] investigated the impact of temperature, relative humidity, and storage time on the electrical properties of smart ultra-high-performance concrete with various functional fillers and free water content by measuring impedance spectra. Zhu et al. [68] employed electrochemical impedance spectroscopy as a NDT method to study the cracking behavior of two types of cementitious materials: engineered cementitious composites and ordinary mortar. Fluctuation observed in the Nyquist plots for $R_{\mathrm{ct}}$ (the impedance caused by charge transfer procedure), along with the stability and smoothness evident in the Bode plot, can be the sensitive indicators of the degree of cracking in the cementitious system. Chung et al. [69] proposed a real-time prediction model for the 28-day compressive strength of concrete at microwave frequencies based on effective conductivity. Mendes et al. [70] proposed an empirical ER model based on commonly used parameters in concrete mix proportions, including aggregate content, water–cement ratio, compressive strength, and cement content. The results of these research indicated that ER could be a performance parameter for the research on novel concrete.

### 3.3. Structural Damage Assessment

The concrete ER is not only closely related to structural strength, but it also changes with the degree of damage. Researchers have studied the relationship between the amplitude and frequency domain of concrete currents and the evolution of cracking. Kyriazoopoulos et al. [71] observed changes in the current signal proportional to the strain rate through conducting uniaxial and three-point bending experiments. The technique offered the potential for in situ evaluation of loading and remaining strength in concrete structures. Fursa et al. [72] proposed a method for evaluating damage in concrete under uniaxial compression based on electrical response to mechanical impacts. Further analysis of the frequency changes in the electrical response under elastic impact excitation was conducted [73]. The investigation revealed that during the elastic deformation stage of concrete specimens, a notable shift of the electrical response spectrum towards lower frequencies occurs. Moreover, a significant displacement of the centershift of the electrical response spectrum in the high-frequency region indicates the occurrence of early-stage cracks. Triantis et al. [74] demonstrated that under high-stress levels, a multitude of microcracks are present, and the generated current by the pressure attains its peak. Fluctuation in the current can serve as an indicator of crack propagation.

**Figure 5:**
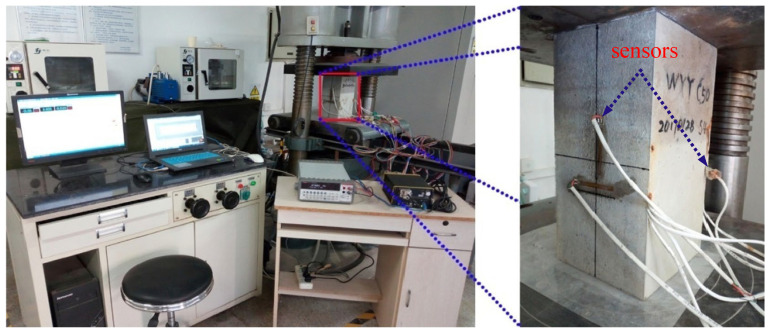
The sensors and experimental platform for monitoring of concrete structures [75]. Copyright 2019, Elsevier.

Through an investigation of the correlation between ER variations and crack development, it becomes feasible to design sensors for the monitoring of concrete structures. Ding et al. [75] successfully integrated ER-based sensors into prefabricated components, thereby creating intelligent building products for practical applications in structural monitoring and calibration. The sensors and the experimental platform are shown in Figure 5. Changes in sensor signals can reflect the stability of concrete structures. Similarly, Amarteja et al. [76] proposed the utilization of embedded piezoelectric sensors to detect the initiation and propagation of localized cracks in concrete while quantifying the alterations in stress wave patterns induced by concrete cracking through a self-compensating attenuation factor.

By integrating concrete ER changes and development of ER sensors, it was possible to conduct quantitative analysis of structural damage. Zeng et al. [77] investigated the correlation between concrete ER and compressive damage, employing the electrode method and the UT method. They established a mathematical relationship between ER and the concrete damage variable. ER results can be affected by considerable uncertainties attributable to various factors, including the water/cement ratio of the concrete and the curing conditions of the structure along with their intricate interconnections. To address this, Dong et al. [78] proposed a concrete SHM and prediction model that incorporates various influencing factors using the XGBoost algorithm. The model provides a reliable and intelligent method to normalize the observed ER results to values under reference conditions. It can also be used for predicting and assessing the durability of concrete structures. Furthermore, Hallaji et al. [79] conducted resistive impedance tomography with large-area surface sensors for the detection of concrete structural damage.

The ER monitoring method offers a valuable approach for the detection and quantitative evaluation of microcracks in concrete structures, when the detection environment is relatively stable. Due to the close correlation between ER and material characteristics, it can be applied in the design of new concrete materials. This method boasts several advantages, including its simplicity in terms of required equipment and minimal human resources for monitoring. However, the ER measurements can be affected by various factors, including temperature, humidity, and electrode contact quality. To achieve accurate ER data, careful attention must be given to electrode arrangement and ensuring optimal contact quality, which ultimately ensures signal stability and reliability. In practical engineering applications, the concrete ER monitoring method is often employed in combination with other monitoring techniques to enhance the comprehensiveness and accuracy of the assessment process.

## 4. Electromagnetic Radiation Monitoring

### 4.1. Introduction and Principle of the EMR Monitoring

**Figure 6:**
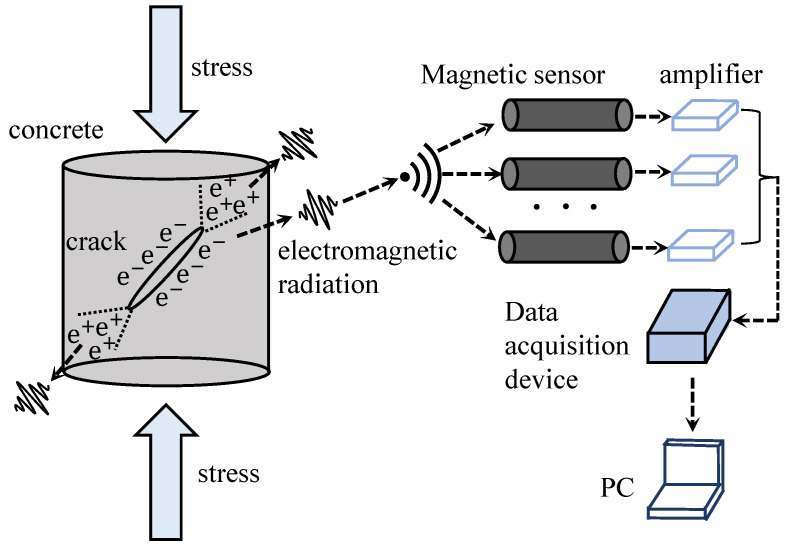
The principle of EMR monitoring.

When concrete undergoes the process of cracking, the surfaces of the cracks experience minute displacements and deformations. As shown in Figure 6, these subtle changes in displacement and deformation cause alterations in stress and charge distribution within the concrete, consequently leading to the generation of an EMR field in the surrounding space. The concept of EMR from brittle materials was initially introduced by Cohen in 1914 [80]. The observation of EMR induced by material fracture was subsequently made by Stepanov in 1933, during experiments involving the application of loads to KCl specimens [81]. In the 1990s, some researchers discovered electromagnetic radiation signals during the concrete cracking process [61].

The phenomenon of EMR generated by concrete fracture has gained significant attention, although the mechanism behind EMR signal generation is not yet fully understood. In the early stages of research on EMR phenomena, several hypotheses and models were proposed by different scholars to elucidate the origin of these signals. These models encompassed various mechanisms, including the piezoelectric effect [82], movement of conductive particles [83], discharge of free charges [84], displacement of moving charges [85], frictional effects [86], rotational vibration of charges [87], and numerous other models, each providing partial explanations for the observed EMR phenomenon. The hypothesis models of EMR phenomena are shown in Table 3. The cracking process of concrete structures is complex, and there is poor correspondence between the micro-mechanisms and macroscopic cracks. It is challenging to quantitatively determine which hypothetical model is specifically applicable through experiments.

**Figure 7:**
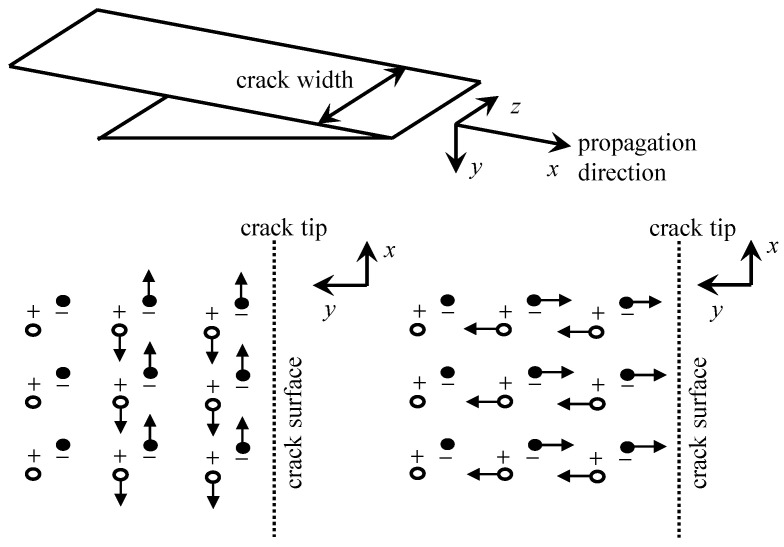
Crack propagation and surface wave oscillation model.

With the progressive advancement of charge generation models, researchers have conducted hypothesis testing to explore the mechanisms underlying charge motion and the subsequent generation of EMR. Kumar et al. [88] detected EMR signals from cubic specimens of cement mortar during quasi-static compression and impact loading processes. It is postulated that the vibration of dipoles formed by ions present in the capillaries and gel pores of cement under impact may contribute to the observed radiation. Han et al. [89] proposed that the EMR originates from variations in charge density induced by the transient electric dipole moment at the crack tip. Ogawa et al. [90] put forth a capacitor-like model to explain charge accumulation on the crack surfaces. O’Keefe et al. [83], based on the literature [90], suggested that the flow of current along the crack tip contributes significantly to restoring charge equilibrium. Among these models, Frid et al. [91,92] developed a surface wave oscillation model for brittle material cracking after years of extensive research. This model aligns well with actual results and is currently widely accepted by the majority of scholars, which is shown in Figure 7. According to this model, when atomic bonds rupture at the crack tip, excited-state atoms oscillate perpendicular to the crack direction and move in tandem with surrounding atoms, generating surface oscillatory waves. This model is independent of material properties, loading modes, and failure modes, and it can theoretically account for the directional characteristics of EMR. The signal’s semi-empirical equation is given in Equation (4).

(4) $$\begin{array}{c} A=\left\{\begin{array}{cc} A_{0}\sin\left(\omega \left(t-t_{0}\right)\right)\left(1-e^{-\frac{t-t_{0}}{\tau }}\right)\; & t<T\\ A_{0}\sin\left(\omega \left(t-t_{0}\right)\right)e^{-\frac{t-T}{\tau }}\left(1-e^{-\frac{T-t_{0}}{\tau }}\right)\; & t\geq T \end{array}\right., \end{array}$$

where *t* represents time, $t_{0}$ is the time from the origin to the start of the pulse, and *T* is the time from the origin to the maximum of the EMR pulse envelope. Therefore, $T-t_{0}$ is the time interval to reach the pulse maximum, τ denotes both the pulse rise time and fall time, and these are considered identical within experimental uncertainty. Additionally, ω stands for frequency, and $A_{0}$ represents the peak amplitude of the pulse.

The superposition model of oscillating dipoles was proposed based on the surface wave oscillation model [93]. This model decomposes the electromagnetic signals generated from fractures into several electromagnetic fields produced by the forced oscillation and damped oscillation of dipoles in different directions and frequencies, which can effectively explain the EMR signals generated when a large number of cracks rapidly occur.

### 4.2. Structural Properties Estimation

The intensity of EMR signals emitted from concrete is closely linked to its mechanical strength. The amplitude of electromagnetic radiation signals is associated with the mechanical properties of concrete, including compressive strength and bonding strength between reinforced cement and aggregates. These EMR signals can be utilized for detecting crack initiation, crack propagation, and mechanical strength of concrete structures.

Some researchers have investigated the relationship between EMR signals of concrete under high temperatures and concrete properties, aiming to identify predictive information for concrete cracking. A multi-field model was developed using COMSOL Multiphysics software to simulate the microwave heating process of single-particle aggregate concrete specimens [94]. The study extensively discussed the evolution of electrical, temperature, stress fields, and moisture transformation during microwave heating. Li et al. [95] studied the EMR characteristics of concrete specimens exposed to high temperatures. The research revealed that as the temperature surpasses 100 degrees, the compressive strength of the specimens decreases while low-frequency EMR signals within the frequency range of 10.9 kHz to 131 kHz emerge. The main frequency and corresponding maximum amplitude of EMR signals increase with rising temperature.

Numerous scholars have also investigated the relationship between EMR signals of concrete and loading conditions as well as material ratios. Fursa et al. [96] focused on investigating the variations in acoustic and electromagnetic emission parameters, identifying high-amplitude AE and the appearance of EMR signals as primary diagnostic criteria for concrete cracking initiation. Li et al. [97] observed that the cumulative EMR counts for concrete, and similar rock-like materials exhibit a decreasing-then-increasing trend with increasing stress levels during loading and unloading. The drastic change in EMR occurs only during collapse. Regarding material proportions, the failure of high-strength polypropylene fiber lightweight concrete under mechanical loading was monitored [98]. Additionally, the EMR response of cement-BaTiO3 (BT) composite materials under impact loading was investigated [99]. The study demonstrated that as the BT content in the composites increased, the volume density, dielectric constant, and piezoelectric charge coefficient of cement-BT materials also increased, while the loss tangent decreased. The EMR response of all cement-BT composite materials showed a direct proportionality to impact height, indicating the effective utilization of EMR monitoring for structural health assessment.

### 4.3. Structural Damage Assessment

The mechanical behavior of concrete and rock specimens under loading until failure has been extensively investigated using AE and EMR analysis. Throughout various studies, it has been consistently observed that AE signals are present during the damage process, while magnetic signals typically arise during rapid stress reduction or final collapse [100,101]. However, some researchers have also discovered that EMR can be observed not only during the material’s failure process but also under impact loading for ceramics, mortar, and concrete [102]. Song et al. [103] conducted a study on the fracture process of rock, coal, and concrete specimens, focusing on potential signals. They found that the variation in surface potential is attributed to the generation of free charges during the material’s failure, which exhibits a strong correlation with the deformation and fracture process of the specimens.

The mechanism underlying the generation of EMR signals during concrete cracking remains not entirely clear. It is the outcome of the synergistic action of multiple factors. Yin et al. [104] conducted experimental studies and observed that concrete generates a magnetic field during the failure process under applied loads. The variation in magnetic induction intensity was found to correspond to the applied load, exhibiting a strong correlation with the AE signal. Consequently, it is postulated that the magnetic field generated during concrete failure is a result of piezoelectric effects, crack propagation effects, and friction effects. Qiu et al. [105] proposed that the change in the EMR signal is significantly influenced by the fracture strength of concrete. The EMR around concrete specimens shows a positive correlation with the applied load. During the constant load phase, the EMR around concrete remains relatively stable, indicating the absence of significant damage. In the elastic deformation stage, where the concrete specimen undergoes load-induced changes, the gradual increase in EMR can be attributed to the piezomagnetic effect, while the rapid increase during the fracture stage is attributed to the friction effect. However, studies indicate that the EMR signals resulting from concrete cracking are positively correlated with the change in load rather than the load itself [106]. Quasi-brittle materials such as rock and concrete with different mechanical behaviors were investigated, and it was observed that EMR accompanied the stress reduction process. The intensity of EMR was found to be related to the magnitude of stress reduction, with a stronger EMR corresponding to greater stress reduction. Furthermore, the amplitude of EMR was approximately proportional to stress reduction, indicating a clear relationship between stress reduction and the EMR signal.

EMR technology can be employed in the creation of damage monitoring sensors. Amit et al. [107] conducted a study on cement mortar/lead zirconate titanate composite materials, focusing on their EMR characteristics when subjected to drop hammer impact. The investigation aimed to explore the potential application of these materials as sensors in civil structures. In a related study, Ai et al. [108] employed a high-resolution industrial camera and a real-time geophysical acquisition system to simultaneously capture microseismic and EMR signals during the crack process. To quantitatively describe the dynamic propagation process of surface cracks in concrete, a robust crack extraction algorithm based on digital image processing was proposed. The findings revealed that cracks propagating parallel to the loading axis exhibited faster propagation rates compared to cracks propagating perpendicular to the loading axis.

The EMR monitoring is a non-destructive method that holds great potential for assessing concrete cracks due to its characteristics of non-contact and remote monitoring. This technique involves the measurement and analysis of EMR signals emitted during the cracking process, enabling the extraction of valuable information regarding crack occurrence, position, and size. However, the mechanism behind the electromagnetic radiation signals generated by concrete cracking remains unclear, and these signals are extremely weak, rendering them highly susceptible to interference from EMR noise in the environment. And certain limitations may arise when dealing with complex concrete structures or attempting to detect small cracks. As a result, the issue of identification and filtering out of spatial EMR noise, enhancing the signal-to-noise ratio of EMR signals, is a future development direction; leveraging of the complementary advantages of EMR and AE methods to achieve the monitoring of concrete structural damage in various scenarios is also a future research direction.

## 5. Piezoelectric Transducers Monitoring

### 5.1. Introduction and Principle of Piezoelectric Transducer Monitoring

**Figure 8:**
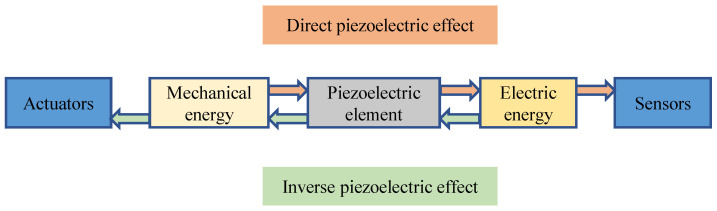
The principle of PZT transducers.

Piezoelectric transducers, constructed from intelligent materials such as PZT (lead zirconate titanate), have found widespread use in smart structural systems. PZT facilitates the bidirectional conversion of mechanical and electrical energy through both direct and inverse piezoelectric effects. The direct piezoelectric effect involves subjecting a piezoelectric element to mechanical vibrations, converting mechanical energy into electrical energy. This effect allows for the development of sensors. Conversely, the inverse piezoelectric effect entails applying voltage to a piezoelectric element, converting electrical energy into mechanical energy. This effect enables the creation of actuators [109]. The operational principle of PZT transducers is illustrated in Figure 8.

In PZT monitoring, wave propagation (WP) and electro-mechanical impedance (EMI) technologies stand out as two commonly employed sensing techniques. In WP technology, the generation and reception of signals involve two types of sensors. Mechanical waves are induced in the material by applying pulses through an actuator, with the resulting electrical signal converted into a propagating wave. The receiving sensor, positioned at a defined distance from the actuator, assesses concrete damage by comparing the time and amplitude decay of wave propagation. The WP technique is founded on the physical correlation between the velocity of R-waves and the wave modulus of elasticity (WMoE) in the propagating medium. Derived from the classical theory of elasticity, the governing equations of Navier are [110]

(5) $$\begin{array}{cc} \left(\lambda +\mu \right)\nabla \nabla \cdot \bar{u}+\mu \nabla^{2}\bar{u} & =\rho \ddot{\bar{u}}, \end{array}$$

where λ and μ are the Lame constants.

The correlation between the velocity of bulk waves and the WMoE in the propagating medium can be represented as [110]

(6) $$\begin{array}{cc} c_{L} & =\sqrt{\frac{\lambda +2\mu }{\rho }}=\sqrt{\frac{E\left(1-v\right)}{\left(1+v\right)\left(1-2v\right)\rho }}, \end{array}$$

(7) $$\begin{array}{cc} c_{T} & =\sqrt{\frac{\mu }{\rho }}=\sqrt{\frac{E}{2\left(1+v\right)\rho }}, \end{array}$$

where $c_{L}$ is L-wave velocity, $c_{T}$ is S-wave velocity, *E* is the WMoE, *v* is Poisson’s radio and ρ is the density of the propagating medium.

**Figure 9:**
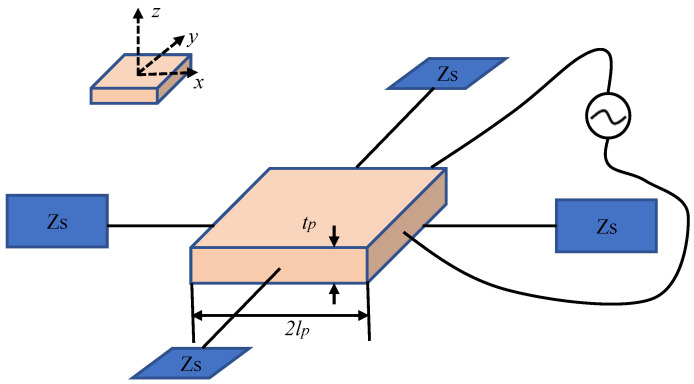
The principle of PZT monitoring.

In EMI technology, a single sensor serves the dual function of emitting and receiving signals. Potential defects or damage are identified by measuring impedance using an impedance analyzer. An interactive model of PZT sensors and concrete structure is shown in Figure 9. The constitutive equations of a PZT sensor, with a length of $2l_{p}$ and a thickness of $t_{p}$, are expressed as [111]

(8) $$\begin{array}{cc} S_{x} & =\frac{1}{\bar{E}}\left(T_{x}-\mu_{p}T_{y}\right)+d_{31}E, \end{array}$$

(9) $$\begin{array}{cc} S_{y} & =\frac{1}{\bar{E}}\left(T_{y}-\mu_{p}T_{x}\right)+d_{31}E, \end{array}$$

(10) $$\begin{array}{cc} D & =\varepsilon_{33}E+d_{31}T_{x}+d_{32}T_{y}, \end{array}$$

where $S_{x}$ and $S_{y}$ are strains in *x* and *y* directions, respectively; $T_{x}$ and $T_{y}$ are stresses in *x* and *y* directions, respectively; $\bar{E_{p}}=E_{p}\left(1+j\eta \right)$ is complex Young’s modulus of PZT sensors; η is the mechanical loss factor; $\varepsilon_{33}=\varepsilon \left(1-j\delta \right)$ is the dielectric constant at zero stress; *D* is electric displacement; δ is the dielectric loss factor; $d_{31}$ and $d_{32}$ are the piezoelectric constants in *x* and *y* directions, respectively; and $\mu_{p}$ is Poisson’s ratio.

SHM based on PZT has been initiated over the past two decades, with the initial focus predominantly centered on damage identification in metal and composite material structures. Soh et al. [112] and Park et al. [113] first demonstrated experimental implementations for damage detection in concrete structures in 2000. In 2003, Bhalla et al. [114] introduced a novel method for damage diagnosis based on changes in high-frequency structural mechanical impedance, utilizing both the real and imaginary parts of the admittance characteristics. The first researchers who employed smart aggregate PZT sensors for monitoring concrete strength observed a reduction in signal amplitude as concrete strength increased [115]. Yang et al. [116] employed structural mechanical impedance extracted from the PZT electromechanical admittance characteristics as a damage indicator, replacing the electromechanical admittance indicator. This substitution further enhances the sensitivity of the system. The key finding in the development of PZT monitoring methods is shown in Table 4.

### 5.2. Structural Properties Estimation

EMI technology is effective in early strength monitoring and durability assessment, serving as a complement to WP technology [119]. Comparing the impedance spectrum of concrete with stress–strain curves, Pan et al. [120] investigated the stress–strain behavior of concrete monitored by PZT sensors and piezoelectric cement (PEC) sensors. Both piezoelectric sensors are applicable for assessing the stress and strain characteristics of concrete. Wang et al. [121] conducted a frequency analysis of smart aggregates embedded with PZT sensors to study the early strength of cement mortar, establishing a linear relationship between strength and resonance frequency. Tang et al. [122] introduced the pioneering on-site application of EMI and WP technology for monitoring concrete curing. Smart aggregate sensors were embedded in the concrete pouring strips of a multi-story residential building during the construction phase. Yu et al. [110] conducted a combined numerical and experimental study using surface-bonded PZT transducers to evaluate the WMoE of fully cured concrete. The generalization and monitoring accuracy of WP methods employing embedded piezoelectric transducers in concrete heavily depend on the driving and sensing mechanisms. Yu et al. [123] investigated the driving and sensing mechanisms of tension-mode piezoelectric transducers. Theoretical analysis results indicate significant differences between sensing and driving mechanisms, influenced not only by piezoelectric constants and transducer dimensions but also by the placement angle of piezoelectric patches and the type of stress wave acting on the sensor. Building upon the experimental data and theoretical modeling, the one-dimensional simplified analytical model grounded in the piezoelectric elasticity theory, explaining the correlation between PZT sensors and cement specimens under uniaxial compression loading, is established [11].

### 5.3. Structural Damage Assessment

WP technology demonstrates potential in damage detection, particularly under compressive, bending, and tensile loads [119]. To address the challenges posed by complex environmental influences and the difficulty of deploying on-site sensors and systems for sustainable, long-term monitoring of concrete structures, the development of an implantable sensor for concrete crack identification is investigated [124]. A multi-sensor integrated concrete implantable module with the appearance of a wall socket is designed to provide stress wave scanning capability within concrete. Ai et al. [125] indicated that electromechanical admittance features exhibit a dual dependency on temperature and heating time, adversely impacting structural damage detection outcomes. These findings promote the inclusion of a time factor when evaluating the influence of temperature on PZT concrete structural monitoring. Smart aggregates have tremendous potential in monitoring concrete structure cracks. Due to the concealed nature of the layered interface, it is challenging to observe or assess the cohesive failure between two components through the development of cracks. Utilizing a two-stage monitoring approach involving the energy attenuation index and damage extent index, Jiang et al. [126] effectively identified the varying degree of cracks on the laminated interface.

Numerous studies in the literature have also explored diverse methods for localizing damage through the utilization of multiple PZT sensors. Gayakwad at al. [111] introduced EMI-WP through synchronized activation of EMI measurements and wave stimulation, enhancing the effectiveness of surface-scanning unit patches in detecting near-field and far-field structural damage. A surface-scanning unit is employed as an electromagnetic interference admittance sensor for localized damage identification. A hybrid algorithm utilized fast discrete wavelet transform, energy methods, and time-of-flight criteria to locate single and multiple damage issues within concrete plates [127]. The proposed method could be applied to localize damage in concrete plates of arbitrary geometric shapes. Liang et al. [128] employed time reversal of the stress wave field in concrete beam specimens, focusing on the crack region, and ultimately identified the damaged areas by accumulating the distribution of energy at each time step. But when there are fewer embedded PZT transducers, spatial resolution decreases. To address this issue, Gao et al. [129] proposed an improved distributed acoustic sensing imaging method with adjustable spatial resolution based on multi-wavelet decomposition.

The state assessment method based on PZT sensors possesses advantages such as low power consumption, ease of manufacturing and installation, suitability for in situ applications, etc., overcoming the shortcomings of traditional monitoring techniques. Additionally, the state assessment method based on PZT sensors demonstrates significant strengths, including real-time and in situ monitoring, high linearity, broad frequency excitation and response, as well as the potential integration with smart structures. Therefore, piezoelectric transducers made of smart materials such as PZT have been extensively utilized in smart structural systems. While it is necessary to deploy a significant number of PZT sensors in large-scale construction environments to monitor concrete strength during the curing period, their proper arrangement, positioning, and spacing can reduce installation time, enhance monitoring efficiency, and save costs. In the case of wired monitoring technology in large concrete projects, the placement of coils connected to PZT sensors has proven challenging, potentially affecting the aesthetic appeal of structures or hindering prolonged monitoring of any strength changes. Therefore, a challenge faced in the application of EMI and WP methods in engineering is to find a successful transition from wired to wireless technology.

## 6. Ultrasound Testing

### 6.1. Introduction and Principle of the UT Method

**Figure 10:**
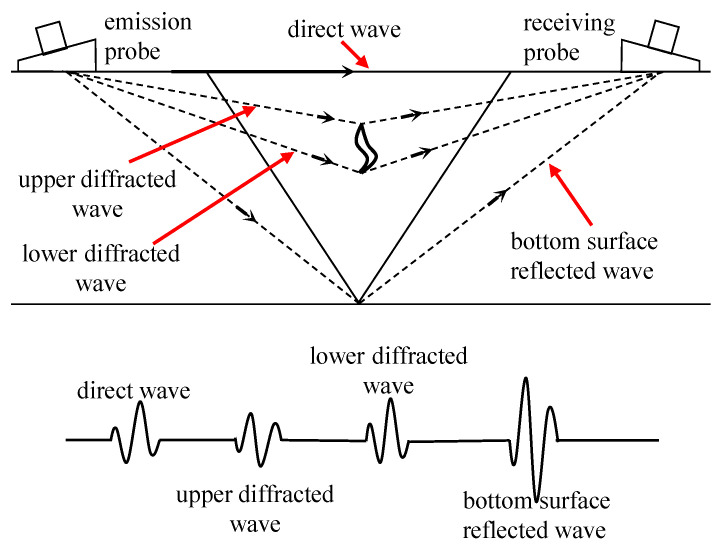
The principle of the UT method.

The fundamental principle underlying the concrete UT method revolves around the propagation and reflection characteristics of ultrasound waves within concrete. As ultrasound waves propagate through concrete structures, they encounter cracks or defects, leading to the reflection of a portion of the energy and the formation of echo signals. Through the measurement and analysis of these echo signals, valuable information regarding the precise location, dimensions, and morphology of cracks can be gleaned. The principle of the UT method is shown in Figure 10.

Similar to AE signals, ultrasonic waves propagating through concrete media are primarily influenced by two types of attenuation: geometrical diffusion and energy dissipation. The theoretical investigation of UT is mainly divided into ultrasonic pulse velocity (UPV) and ultrasonic pulse amplitude (UPA) methods. UPV can be written as [130]

(11) $$\begin{array}{cc} V_{c}\left(x,t\right) & =\frac{x}{t}, \end{array}$$

where $V_{c}\left(x,t\right)$ represents the UPV in concrete, with *x* being the propagated path length and *t* being the transit time. The relationship between $V_{c}\left(x,t\right)$ and the compressive strength of concrete $f_{c}$ is [131]

(12) $$\begin{array}{cc} f_{c} & =ae^{bV_{c}}, \end{array}$$

where *a* and *b* are parameters that depend on material properties.

The relative amplitude ratio of UPA, $A_{r}$, is defined as the ratio of the reflected wave amplitude to the original amplitude [130].

(13) $$\begin{array}{cc} A_{r}\left(x\right) & =\frac{P_{x}}{P_{o}}=K_{c}K_{d}\frac{1}{x}e^{-\alpha x}, \end{array}$$

where $P_{x}$ represents the pulse amplitude at distance *x* from the source, $P_{0}$ is the initial pulse amplitude at the source, $K_{c}$ is the attenuation factor attributed to contact losses, $K_{d}=\pi \left(D_{0}^{2}/4\delta \right)$ is the geometrical divergence coefficient for the material, $D_{0}$ is the oscillator’s diameter, δ is the wavelength of the sound beam, and α represents the attenuation coefficient.

The origin of the concrete ultrasound method can be traced back to the 1960s and 1970s when ultrasound technology was introduced to the realm of concrete engineering as a means of non-destructive testing and structural evaluation. In the 1990s, elastic waves within the ultrasonic frequency range were widely applied for non-destructive assessment of defects in concrete. In the 21st century, there has been a gradual increase in the hybrid monitoring approach combining ultrasound with AE and IRT. Nowadays, the UT method is n one of the most researched and widely spread methods of NDT.

### 6.2. Structural Property Estimation

Ultrasound signals, in their transmission path, exhibit a close correlation with material properties, making them capable of assessing the strength of concrete structures. Fontoura et al. [132] utilized embedded ultrasonic transducers along with temperature and humidity sensors to monitor concrete specimens and real structures. However, several factors, which may not exert the same influence on concrete compressive strength, could impact the experimental UT values differently. To address this issue, Silva et al. [133] explored the potential of estimating compressive strength through an artificial neural network by considering pertinent parameters such as water–cement ratio, aggregate–cement ratio, testing age, and cement-to-metakaolin ratio. The velocity of ultrasound waves correlates with material stiffness, and wave attenuation can be employed to assess the condition of damaged concrete structures. J. Sokolowska et al. [134] investigated the impact of utilizing polyethylene terephthalate (PET) instead of quartz on ultrasound propagation in polymer cement concrete (PCC). The ultrasonic velocity is highly correlated with flexural strength and compressive strength. Yim et al. [135] established statistical correlations between extracted parameters from received ultrasonic profiles and mechanical properties. Liu et al. [136] conducted comprehensive laboratory experiments to establish correlations between ultrasonic pulse velocity (UPV), porosity, and compressive strength, employing UPV experiments and compressive strength tests as a reference for utilizing ultrasound in monitoring the mechanical properties of concrete in civil engineering practice.

### 6.3. Structural Damage Assessment

Conventional NDT methods face challenges in monitoring internal damage in concrete. But ultrasound signals have proven to be effective in assessing the extent of internal microcracks in concrete structures [15]. Ham et al. [137] proposed a method to characterize the volume content of relatively small distributed microcracks in concrete using ultrasonic surface wave backscattering measurements. Wang et al. [138] synchronized the monitoring and characterization of damage, specifically microcracks, in concrete specimens during multiple loading steps using both active ultrasound and passive AE techniques, revealing that damage evolution is not only stress-dependent but also time-dependent.

In practical engineering applications, cracks typically emerge from the interplay of various factors, giving rise to intricate patterns in the cracking region. To address the challenge of detecting complex cracks, Sami et al. [139] developed mathematical models to automate the interpretation of ultrasonic measurements. Niu et al. [140] applied Bayesian theory to combine travel time and wave attenuation for evaluating internal defects in concrete structures, enabling comprehensive fault scanning without the need for additional measurements. Zhao et al. [141] considered the effects of loading and microcracks on diffused waves and proposed a decorrelation model based on sensitivity kernels, which was successfully applied to concrete beams to identify the locations and depths of multiple existing cracks. Ahn et al. [142] proposed a concrete micro-crack damage assessment method that combines the ultrasonic wave technique with air-coupled sensing. This approach significantly reduces data collection time while maintaining data reliability.

**Figure 11:**
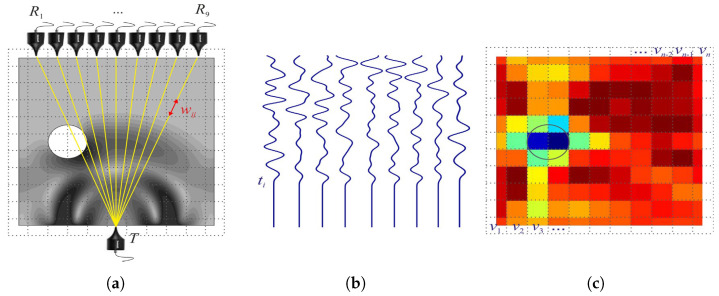
Schematic diagram of ultrasonic transmission tomography: (**a**) cross-section divided into pixels, with indicated transmitter, receivers and simulated wave field, (**b**) wave propagation signals, and (**c**) tomographic reconstruction image (The circles indicate the locations of defects) [146] (Copyright 2020, Elsevier).

In addition to damage detection and quantification, UT methods offer the capability to image the cracked areas in concrete. Zhao et al. [143] employed piezoelectric ceramic-induced ultrasound and the time reversal method to locate and characterize defects along the reinforced concrete interface, enabling the imaging of defects through cross-sectional scans. Zielinska et al. [144] utilized ultrasonic tomography to visualize the internal structure of tested components by employing a novel method to determine the flight time of waves from the transmitter to the receiver. Jia et al. [145] utilized ultrasound-excited thermal imaging to detect microcracks in concrete materials. This technique effectively identified concrete cracks with widths ranging from 0.01 to 0.09 mm. Monika et al. [146] developed a new theoretical model to determine the paths of transmitted, refracted, and reflected elastic waves, as well as creeping waves propagating along the inclusion surface. The schematic diagram of ultrasonic transmission tomography is shown in Figure 11.They successfully imaged the internal structure of the tested beam based on wave propagation measurements on its surface and computer tomography scans. The results demonstrated that ultrasound tomography holds significant potential for detecting debonding in reinforced concrete structures.

The method for locating damage positions using ultrasonic waves primarily relies on establishing an ultrasonic decay model to determine them. Based on the decay patterns of ultrasonic waves, Ewald et al. [147] proposed an adaptability function. Through various algorithms, a global structural health monitoring sensor placement strategy was specified to balance the requirements of detecting predetermined and randomly occurring damage locations. By investigating the differences in ultrasonic decay ratios at different positions, Yu et al. [148] proposed a model that successfully quantifies the attenuation of reflection signals at various crack locations. Additionally, the accuracy of crack localization was enhanced through the incorporation of an improved elliptical positioning algorithm.

UT testing is a reliable method for the extensive monitoring of concrete. It is capable of imaging and locating defects, representing a mature technology with a range of products already implemented in industrial production. However, it is crucial to acknowledge that the material properties of concrete, including sound velocity, attenuation, and other relevant parameters, exert an influence on the propagation and reflection of ultrasonic waves. Therefore, it becomes essential to perform proper calibration and correction procedures tailored to the specific characteristics of different concrete materials. Furthermore, it is important to note that UT methods typically involve measurements conducted on or in proximity to the surface of the concrete. Consequently, challenges may arise when attempting to monitor concrete structures that are deeply embedded or have limited accessibility.

## 7. Infrared Thermography Monitoring

### 7.1. Introduction and Principle of the IRT Method

**Figure 12:**
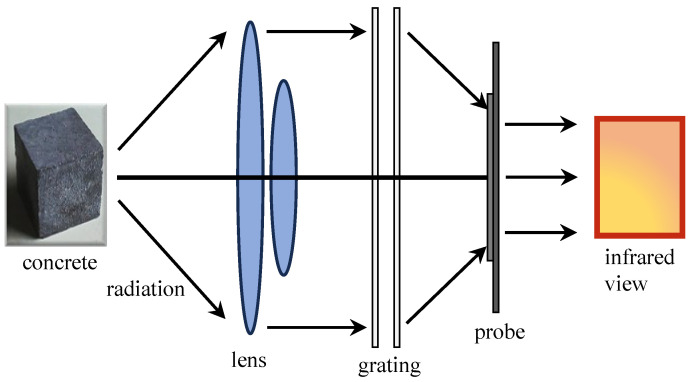
The principle of IRT monitoring.

The IRT monitoring of concrete is a detection method that relies on IRT technology to evaluate the cracking and damage of concrete structures. This approach capitalizes on the distinctive radiation properties of concrete materials within the infrared frequency range. When concrete structures undergo cracking or damage, alterations in their thermal conductivity occur, leading to localized temperature variations and generating distinct IRT signals within the concrete material. The principle of IRT monitoring is shown in Figure 12.

Different thermal imaging techniques can yield a variety of thermal response patterns. The most common thermal responses in IRT are the thermal signal ΔT and thermal contrast *C* [149]. Time-dependent thermal signal $\Delta T\left(t\right)$ can be calculated from Equation (14),

(14) $$\begin{array}{cc} \Delta T\left(t\right) & =T{\left(t\right)}_{defect}-T{\left(t\right)}_{background}, \end{array}$$

where $\Delta T\left(t\right)$ represents the thermal signal at specific time *t*, $T{\left(t\right)}_{defect}$ is the surface temperature recorded above the defect at that specific time, and $T{\left(t\right)}_{background}$ is the surface temperature recorded in the background where no sub-surface defect is present at that specific time.

Contrast C can be calculated from Equation (15),

(15) $$\begin{array}{cc} C\left(t\right) & =\Delta T\left(t\right)/\left(T{\left(t\right)}_{background}-T{\left(t\right)}_{ambient}\right), \end{array}$$

where $C\left(t\right)$ denotes the thermal contrast at specific time $\left(t\right)$, $\Delta T\left(t\right)$ represents the thermal signal of the defect at that specific time in degrees Celsius, $T{\left(t\right)}_{background}$ is the recorded surface temperature in the surrounding defect-free areas at that specific time in degrees Celsius, $T{\left(t\right)}_{ambient}$ is the ambient temperature, with most of the tests conducted at 20 °C.

IRT, which is founded on infrared technology, has emerged as a geophysical technique for monitoring the instability and deterioration of concrete. By converting invisible infrared patterns into visual images, known as infrared images, IRT enables the identification of defects in concrete structures by analyzing differences in temperature distribution. IRT not only captures the temporal characteristics of thermal images, but also provides valuable spatial information within the IRT field. It can be utilized for layered detection in concrete. Ta et al. [149] provided a detailed introduction to the detection of various debonding, delamination, and layered areas within the bond region of single and multiple layers of carbon fiber reinforced polymer (CFRP) with concrete structures. The results indicate that the maximum thermal signal is directly proportional to the number of CFRP layers. Hiasa [150] studied the influence of the size of delaminations (area, thickness, and volume), environmental temperature, solar irradiance conditions (different seasons), and the depth of delaminations from the surface on the results of delamination. This study indicated that the influence of layered areas on the detectability of IRT was far greater than that of thickness and volume. It was also observed that there were no significant differences based on the season in which IRT was employed. As an emerging technology for monitoring concrete structures, it exhibits minimal sensitivity to composition and stress states. Its primary influences are environmental temperature and inherent resolution. Presently, it is primarily employed for assessing concrete damage.

### 7.2. Structural Damage Assessment

In the early stages, IRT monitoring methods in concrete structures were associated with large errors and provided primarily qualitative information about defect depth, necessitating the use of elastic wave signals for further depth determination [151]. To enhance the accuracy of IRT monitoring methods, Hiasa et al. [152] proposed an automated threshold determination method that combines finite element modeling simulation, facilitating damage confirmation through color contrast analysis of images. Considering the impact of temperature on IRT monitoring, Hiasa et al. [153], through field experiments and finite element modeling simulations, identified nighttime clear-sky conditions as optimal time windows for IRT monitoring of concrete bridge decks.

**Figure 13:**
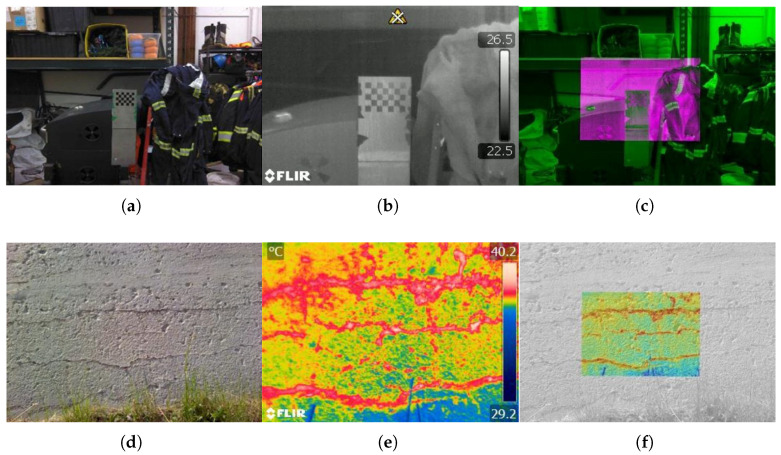
Combining visible and IRT images: (**a**,**b**) Visible and a thermal reference image for calibration; (**c**) Superposition of calibrated reference images (**a**,**b**); (**d**,**e**) Visible and thermal image of concrete structure; (**f**) Superposition of visible and infrared images (**d**,**e**) [155] (Copyright 2022, Elsevier).

Despite technological advancements facilitating the acquisition of thermal images, practical NDT of concrete structures still requires improvements in contrast and resolution. The quantification and differentiation of defects in IRT monitoring methods often rely on the subjective expertise of inspectors, resulting in significant uncertainties. To address this challenge, Jang et al. [154] introduced a deep learning-based hybrid image autonomous concrete crack detection technique. By combining visual and IRT images in hybrid images, crack detectability is enhanced while minimizing false positives. Pozzer et al. [155] investigated semantic segmentation of common concrete defects using various imaging modes. They trained a pre-trained convolutional neural network (CNN) model through transfer learning to detect concrete defects, including cracks, spalling, and potential subsurface defects. The system is shown in Figure 13. In terms of instability and failure of concrete structures, Lou et al. [156] examined the relationship between IRT field distribution, surface morphology features, and stress field distribution. By comparing the similarity of IRT distribution with Gaussian distribution, a significant decrease in similarity was identified as a precursor to concrete structural instability.

As an emerging technology, IRT monitoring offers the advantage of swift surface scanning, facilitating a rapid assessment of crack distribution in concrete structures. It is a method that characterizes the damaged areas of concrete through imaging. Utilizing an infrared camera, the temperature distribution on the concrete surface can be captured, enabling a preliminary evaluation of crack depth and extent. Nonetheless, it is important to acknowledge that IRT monitoring is susceptible to environmental conditions, including variations in temperature and wind speed. These factors can influence heat transfer dynamics and subsequently impact the accuracy of measurement results. Consequently, it becomes crucial to consider and mitigate these environmental effects to ensure the reliability and precision of IRT monitoring in concrete crack assessment.

## 8. Conclusions

This paper presents a comprehensive review of five methods employed in the monitoring of concrete structure properties and damage evolution, encompassing their underlying principles, applications, advantages, and limitations. The results are shown in Table 5. The synthesized research findings presented herein provide a valuable foundation for future investigations and practical applications in the field.

In summary, each monitoring method has its applicability in specific domains, but it also comes with some unavoidable challenges. Among these, as one of the most widely applied technologies, AE monitoring has been utilized in various scenarios, including bridges, concrete beams, and smart concrete design. Through the analysis of time records, arrival time differences, energy levels, and amplitude variations of AE signals generated during concrete cracking, this method facilitates the determination of crack initiation time, quantity, location, and activity. Nevertheless, it is notably impacted by vibrations and lacks the capability for long-distance monitoring. Quantitative techniques for intricate concrete cracks are still in the early stages of development, and the disentanglement of the effects of multiple cracks represents a direction for future advancements.

ER monitoring deduces changes in concrete properties and damage evolution by analyzing variations in electrical resistance. This approach offers several advantages, characterized by its simplicity in terms of required equipment and the minimal human resources needed for monitoring. It has significant applications in concrete strength monitoring and smart concrete design. It is susceptible to environmental influences and material effects, but due to its insensitivity to vibration noise, it is often combined with AE methods to enhance the scope and precision of monitoring. Arrangement of electrodes to ensure contact quality and improve the stability and accuracy of resistivity signals is a key focus for future research.

The mechanism behind EMR monitoring is not fully elucidated, and its signals are prone to interference from EMR noise. As a result, its extensive application is currently confined, primarily finding utility in environments with lower electromagnetic noise, such as tunnels and caves. Nevertheless, owing to its attributes of large-scale, non-contact monitoring, there is substantial potential for advancement if monitoring sensitivity can be improved. As a consequence, the identification and filtration of spatial EMR noise to enhance the signal-to-noise ratio of EMR signals represent a future development direction. Additionally, exploring the synergistic advantages of EMR and AE methods for monitoring concrete structural damage across diverse scenarios is another avenue for future research.

The PZT monitoring method involves embedding or placing piezoelectric sensors on the surface of concrete to monitor the properties and damage evolution of concrete structures. It has advantages such as high monitoring sensitivity and real-time capabilities. However, its monitoring range is limited, and the complex structure of sensor placement makes it challenging for large-area monitoring. Exploring a wireless transmission method to reduce system complexity is a future research direction.

The UT method is widely employed and non-destructive, known for its high precision and long detection range. However, its accuracy is susceptible to the internal pores of concrete, posing challenges in achieving comprehensive monitoring of the entire damage process. It is imperative to conduct meticulous calibration and correction procedures customized for the distinct characteristics of various concrete materials. Additionally, it is crucial to acknowledge that UT methods usually entail measurements performed on or near the surface of the concrete. Consequently, challenges may arise when endeavoring to monitor concrete structures deeply embedded or possessing limited accessibility.

IRT monitoring, as a long-distance monitoring method, offers extensive coverage but exhibits diminished accuracy, being susceptible to environmental influences. It is commonly employed in the monitoring of large-scale structures. Addressing and mitigation of these environmental effects to ensure the reliability and precision of IRT monitoring in assessing concrete cracks is the future research direction.

In the evaluation of concrete properties and the monitoring of damage evolution, the future trend involves the synergistic use of multiple monitoring methods. Among them, the EMR and IRT monitoring methods, with their long-range capabilities and extensive coverage, can serve as preliminary criteria for assessing damage evolution, offering qualitative insights into structural stability. AE, ER and PZT, as local monitoring methods, can concentrate on monitoring relatively severe damage areas, facilitating the quantitative identification of structural risks. UT can assist AE methods in evaluating and analyzing the evolution of deep internal defects in concrete. Combination of various monitoring methods allows for the establishment of a model for the entire process of concrete damage evolution, enabling the assessment of structural stability and the functionality of danger warning.

## Figures and Tables

**Table 1:** The key findings in the development of AE monitoring methods.

Researcher	Key Findings
[24]	The Caesar Effect is present in metals in 1950.
[24]	In the 1960s, the AE method is first applied to concrete testing.
[25]	The *b*-value of acoustic emission signals exhibits consistency with the cumulative damage in concrete in 2003.
[26,27]	Since 2010, the relationship between acoustic emission signals and the cracking behavior of concrete is gradually gaining attention.
[28,29]	The localization of defect positions through the Akaike information criterion method is proposed in 2012.
[30]	After 2016, there has been a growing interest in the relationship between acoustic emission signals and the composition of concrete.

**Table 2:** The key findings in the development of ER monitoring methods.

Researcher	Key Findings
[60]	The measurement of concrete ER began in the 1960s.
[61]	The relationship between concrete ER and stress was established in the 1990s.
[62]	Conductive concrete can serve as a smart structural material, enabling non-destructive electrical probing for defect monitoring in the 1990s.
[63]	Electrical Impedance Tomography can be employed to measure the internal strain field, thereby achieving crack imaging in 2009.

**Table 3:** Hypothesis models of EMR phenomena.

Model Name	Specific Explanation	Limitations
Piezoelectric effect [82]	Quartz crystals in the material generate positive and negative charges under compression	Unable to explain the existence of EMR in materials without quartz crystals
Movement of conductive particles [83]	Cracks indicate the influence of conductivity on the amplitude of EMR signals	Unable to explain the phenomenon of EMR in materials with low conductivity
Discharge of free charges [84]	Rapid discharge of free charges during crack propagation	Unable to explain the phenomenon of EMR in materials with low conductivity
Displacement of moving charges [85]	Crystal dislocations exist, and when stress is applied, dislocations undergo transverse slip	The model underestimates the intensity of the signal
Frictional effects [86]	Friction generates charges during the formation of microcracks	Unable to explain the presence of EMR during the compression process
Rotational vibration of charges [87]	Charged particles undergo rotational vibration during the fracture process	Unable to explain the directional aspect of EMR

**Table 4:** The key findings in the development of PZT monitoring methods.

Researcher	Key Findings
[112,113]	The piezoelectric transducers were used to detect concrete damage.
[114]	Damage diagnosis based high-frequency structural mechanical impedance.
[115,117]	Early-age strength monitoring using embedded piezoceramic transducers.
[118]	Concrete SHM using embedded piezoceramic transducers.
[116]	Damage diagnosis based structural mechanical impedance.

**Table 5:** Comparison of concrete structure properties and damage evolution monitoring methods.

Method	Principle	Applications	Advantages	Limitations
AE	AE signals generated by concrete fracture vibrations	Concrete bridges [6,8,22] Concrete beams [25,34,39,51,55] Smart concrete [32,33]	Simple operation High sensitivity Inner monitoring Real time	Susceptible to vibration effects Local monitoring
ER	ER changes caused by charge movement within concrete	Smart concrete [66,67,75] Concrete beams [62]	Simple operation Inner monitoring Real time	Low sensitivity Susceptible to material effects Local monitoring
EMR	EMR signals generated by charge oscillation during concrete fracture	Smart concrete [98,99]	Simple operation Inner monitoring Non-contact monitoring Real time Remote monitoring	Low sensitivity Susceptible to EMR environment effects
PZT	PZT signals generated by piezoelectric sensors in response to stress	Smart concrete [109,111,122] Concrete bridges [112] Concrete beams [119,128]	High sensitivity Simple operation Inner monitoring Real time	Complex operation Local monitoring
UT	Ultrasonic wave propagation and reflection characteristics within concrete	Concrete bridges [15] Concrete beams [143,146]	High sensitivity Inner monitoring Remote monitoring	No real time Susceptible to concrete pores
IRT	Thermal imaging differences in concrete materials	Smart concrete [149] Concrete bridges [152,153]	Visualization of results Non-contact monitoring Remote monitoring	Low sensitivity Susceptible to temperature effects

## Abbreviations

The following abbreviations are used in this manuscript:

AE	Acoustic emission
CFRP	Carbon fiber reinforced polymer
ER	Electrical resistivity
EMI	Electro-mechanical impedance
EMR	Electromagnetic radiation
IRT	Infrared thermography
NDT	Non-destructive testing
PCC	Polymer cement concrete
PET	Polyethylene terephthalate
PZT	Lead zirconate titanate
UT	Ultrasonic testing
SHM	Structural health monitoring
UPA	Ultrasonic pulse amplitude
UPV	Ultrasonic pulse velocity
WMoE	Wave modulus of elasticity
WP	Wave propagation
